# **Effect of intravenous anesthetic drugs on fertilization rate in oocyte retrieval**

**DOI:** 10.1186/s12871-024-02542-5

**Published:** 2024-04-29

**Authors:** Yanfang Liu, Yongtao Gao, Cuie Lu, Junjie Chen, Saisai Huang

**Affiliations:** Department of Anesthesiology, Affiliated Hospital of Nantong University, No. 20 Xisi Road, Nantong, Jiangsu Province 226001 China

**Keywords:** Fertilization rate, Intravenous anesthetic drugs, Oocyte retrieval, Propensity score matching (PSM)

## Abstract

**Background:**

The purpose of this study was to investigate the effects of intravenous anesthetic drugs on fertilization rate in subjects receiving oocyte retrieval by assisted reproduction technology (ART).

**Methods:**

A retrospective cohort study was designed. The clinical information of subjects who received oocyte retrieval procedure was collected. The subjects were divided into two groups based on the type of anesthesia used: the no-anesthesia group and the intravenous anesthesia group. Propensity score matching (PSM) was performed and multiple linear regression analyses were conducted. Fertilization rate was compared between the two groups before and after PSM.

**Results:**

A total of 765 subjects were divided into two groups: the no-anesthesia group (*n* = 482) and the intravenous anesthesia group (*n* = 283). According to propensity scores, 258 pairs of subjects were well matched, and the baseline data between the two groups were not significantly different (*P* > 0.05). Fertilization rate was 77% in the intravenous anesthesia group, and 76% in the no-anesthesia group, without significant between-group difference (*P* = 0.685). Before matching, Poisson regression analysis showed no effect of intravenous anesthetic drugs on fertilization rate (RR = 0.859, 95%CI: 0.59 to 1.25, *P* = 0.422). After matching, no difference was found either (RR = 0.935, 95%CI: 0.67 to 1.29, *P* = 0.618).

**Conclusion:**

Intravenous anesthetic drugs may exert no effects on fertilization rate in subjects receiving ART.

**Supplementary Information:**

The online version contains supplementary material available at 10.1186/s12871-024-02542-5.

## Introduction

Transvaginal ultrasound-guided oocyte retrieval, a standard but invasive procedure in in vitro fertilization and embryo transfer (IVF-ET), can be performed to obtain viable oocytes from ovarian follicles before ovulation through needle retrieval [1]. As the needle punctures through the vaginal wall and explores the eggs in the ovary, the subjects experience pain, which can be relieved by antiesthetic drugs [2]. However, it is unknown whether intravenous anesthetic drugs affect the fertilization rate and the in vitro fertilization (IVF) outcomes.

During states of general anesthesia, the administration of propofol has been observed to decrease perfusion pressure in both follicles and the endometrium, as well as reduce hemoglobin concentration and plasma cortisol levels. The hemodynamic and biochemical alterations that occur following propofol infusion may have indirect implications for pregnancy outcomes. Propofol, known for its high lipophilicity, exhibited a direct relationship between the cumulative dose of propofol administered and the progressive accumulation of its concentrations in follicular fluid. The accumulation of propofol in follicular fluid has the potential to impact oocyte fertilization and subsequently influence the quality of embryos. Previous animal studies [3, 4] have indicated that propofol may have an impact on early embryonic development, although limited human studies [5] have been conducted. In light of these findings, we sought to investigate the potential effects of propofol exposure during pregnancy.

Therefore, we designed this retrospective cohort study involving subjects matched through PSM. This study attempts to explore the potential effects of intravenous anesthetic drugs on the IVF outcomes of subjects.

## Methods

We retrospectively analyzed the clinical data of subjects who received oocyte retrieval in the Affiliated Hospital of Nantong University from January 2020 to December 2021. The inclusion criteria were as follows: (1) infertile females who received oocyte retrieval in IVF treatment; (2) follow-up data were complete. The exclusion criteria were as follows: (1) the subjects presented comorbidities, including hypertension, diabetes, liver diseases, kidney diseases, thyroid illness and autoimmune diseases; (2) the subjects showed oocyte cryopreservation and no oocyte cycles; (3) the subjects had taken other therapies after IVF. The study complies with the ethical guidelines of the Declaration of Helsinki and was approved by the Institutional Review Board of Affiliated Hospital of Nantong University (No: 2019-K039), and informed consent was obtained from all subjects.

The subjects were divided into the no-anesthesia group and the intravenous anesthesia group. In the no-anesthesia group, the oocyte retrieval was performed in the subject under a waking state. In the intravenous anesthesia group, the oocyte retrieval was performed in the subject falling asleep after anesthesia using intravenous propofol. Subject data including number of IVF cycles, ages of the couple, body mass index (BMI) of the female, duration of infertility, type of infertility (primary, secondary), infertility causes (tubal factor, ovulation disorders, endometriosis, premature ovarian insufficiency [POI], uterine factor, male factor, other causes and unexplained causes), ovarian stimulation protocols (A, B, C, D, E, F, G, H, I), basal follicle-stimulating hormone (FSH), basal luteinizing hormone (LH), basal estradiol (E2), basal antral follicle count (AFC), basal cancer antigen 125 (CA125), launch-day follicle-stimulating hormone (FSH), launch-day luteinizing hormone (LH), launch-day estradiol (E2), launch-day antral follicle count (AFC), trigger-day luteinizing hormone (LH), trigger-day estradiol (E2), trigger-day progesterone (P), the number of oocytes, the number of mature oocytes, fertilization way (IVF, intracytoplasmic Sperm Injection [ICSI], Half Intracytoplasmic Sperm Injection [HALF-ICSI]), anesthetic modality (no-anesthesia or intravenous anesthesia). The primary outcome was fertilization rate. In this study, fertilization rate was defined as the number of fertilized oocytes divided by the total number of retrieved oocytes.

In our center, ovarian stimulation was performed based on the female’s age and ovarian reserve function. (A) The luteal phase long protocol: gonadotropin releasing hormone agonist (GnRH-a) was administrated in the luteal phase of the previous cycle; (B) The follicular phase long protocol: GnRH-a was administrated in the midluteal phase; (C) The ultra-long GnRH-a protocol: women received subcutaneous injections of long-acting GnRH-a for 2 to 4 months. (D) The ultra-short GnRH-a protocol: in this protocol, GnRH-a was used only once on day 2 of menstruation, after which gonadotropin (Gn) was initiated on day 3 and maintained until the administration of HCG. (E) The GnRH antagonist protocol: human menopausal gonadotropin (HMG) was administered daily from menstrual cycle day 3, and GnRH antagonist (0.25 mg/day) was added from stimulation day 6. (F) The progestin-primed ovarian stimulation (PPOS) protocol: hMG at 150–225 IU and medroxyprogesterone acetate (MPA) at 10 mg were administered daily from cycle day 3. (G) The micro-stimulation protocol: clomiphene was given orally from days 2 to 3 of the menstrual cycle. (H) The natural cycle protocol: no ovulation-inducing medication was given. (I) The other protocol: other methods for the treatment. Launch-day was defined as day 3–5 of a menstrual cycle, and trigger-day as the day of ovulation triggered with hCG or GnRH agonists.

**Fig. 1:**
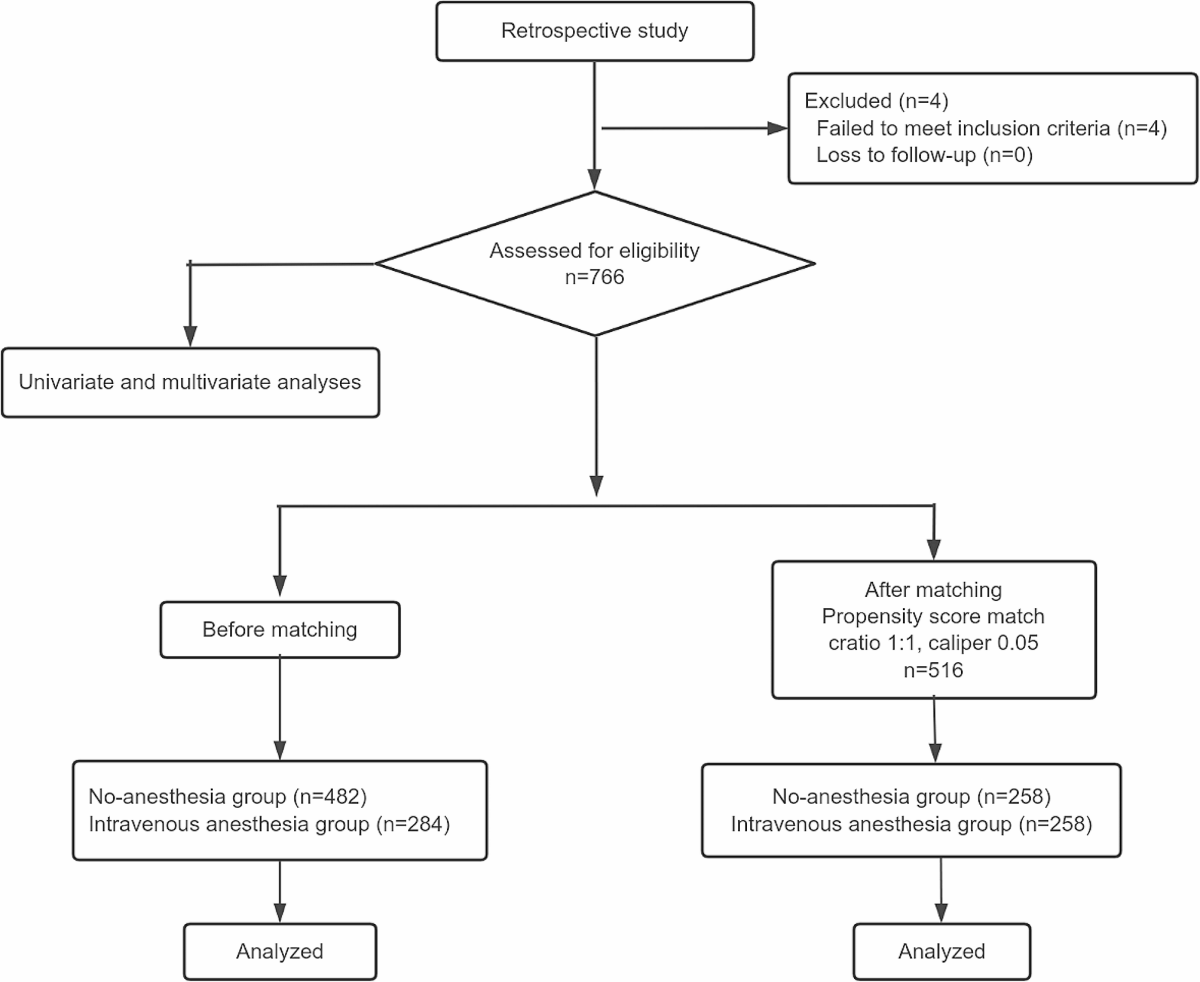
Flow diagram

SPSS 25.0 statistical software was used for analysis. In the study, continuous variables were expressed as mean means ± standard deviation, and compared through Mann-Whitney U test. Categorized data were presented as rate (%), and compared through the Chi-square test. Poisson regression was used for multivariate analysis. A significant difference was considered at *P* < 0.05. The two groups were balanced using PSM. We used 1:1 match on the nearest neighbor, and the caliper value was 0.05 (Fig. 1). A standardized difference of more than 0.1 indicated that the two groups were well balanced. Adjusted covariates in PSM included number of IVF cycles, ages of the couple, BMI of the female, duration of infertility, type of infertility, infertility diagnoses, ovarian stimulation protocols, basal FSH, basal LH, basal E2, basal AFC, basal CA125, launch-day FSH, launch-day LH, launch-day E2, launch-day AFC, trigger-day LH, trigger-day E2, trigger-day P, the number of oocytes, the number of mature oocytes, fertilization method, anesthetic modality.

## Results

Before matching, statistically significant differences were found in the characteristics between the two groups, such as POI factor (*p* = 0.017) and male factor (*p* = 0.038). After matching, the baseline data between the two groups were not significantly different (*P* > 0.05). Before matching, the fertilization rate was 77% in the no-anesthesia group, and 76% in the intravenous anesthesia group, without significant between-group difference (*P* = 0.443). After matching, no difference was observed either (*P* = 0.685) (Table 1).

**Table 1:** Comparison of clinical features before and after matching between the two groups case

Variables	Before matching				After matching			
	N0-anesthesia group(*n* = 482)	Intravenous anesthesia group(*n* = 284)	SMD	*P*	No-anesthesia group(*n* = 258)	Intravenous anesthesia group(*n* = 258)	SMD	*P*
Number of IVF cycles, mean (SD)	1.38 (0.69)	1.36 (0.70)	-0.028	0.702	1.34 (0.67)	1.36 (0.68)	0.028	0.744
Female age, mean (SD), y	31.06 (4.67)	30.56 (4.64)	-0.107	0.154	30.82 (4.43)	30.75 (4.73)	-0.015	0.863
Female BMI, mean (SD), kg/m^2^	23.13 (3.54)	23.15 (3.94)	0.005	0.946	23.13 (3.51)	23.26 (4.01)	0.033	0.693
Male age, mean (SD), y	32.06 (4.21)	31.54 (4.60)	-0.113	0.112	31.77 (3.84)	31.74 (4.69)	-0.005	0.951
Duration of infertility, mean (SD), y	3.20 (2.17)	3.07 (2.05)	-0.066	0.399	3.25 (2.12)	3.11 (2.07)	-0.070	0.440
Basal FSH, mean (SD), IU/L	7.75 (2.50)	7.46 (2.26)	-0.126	0.116	7.59 (2.32)	7.54 (2.30)	-0.022	0.805
Basal LH, mean (SD), IU/L	4.67 (2.50)	4.84 (2.76)	0.062	0.379	4.76 (2.62)	4.82 (2.75)	0.022	0.797
Basal E2, mean (SD), pg/ml	42.65 (19.16)	42.66 (22.05)	0.000	0.995	42.61 (19.84)	42.80 (21.99)	0.008	0.919
Basal AFC, mean (SD)	17.55 (11.95)	18.92 (12.47)	0.110	0.131	19.29 (12.32)	18.52 (12.17)	-0.062	0.472
Basal CA125, mean (SD), U/ml	15.32 (6.81)	14.77 (6.64)	-0.082	0.280	14.94 (6.63)	14.64 (6.62)	-0.045	0.607
Launch-day FSH, mean (SD), IU/L	7.76 (2.64)	7.47 (2.49)	-0.115	0.140	7.56 (2.56)	7.55 (2.52)	-0.004	0.960
Launch-day LH, mean (SD), IU/L	4.61 (2.76)	4.67 (2.85)	0.020	0.784	4.60 (2.76)	4.69 (2.85)	0.031	0.723
Launch-day E2, mean (SD), pg/ml	46.87 (51.83)	44.66 (37.66)	-0.059	0.496	47.09 (57.39)	44.71 (38.79)	-0.063	0.583
Launch-day AFC, mean (SD)	14.15 (8.79)	15.04 (8.37)	0.107	0.168	14.86 (8.82)	14.94 (8.46)	0.009	0.919
Trigger-day LH, mean (SD), IU/L	3.81 (3.15)	3.65 (2.80)	-0.059	0.455	3.62 (2.92)	3.75 (2.84)	0.047	0.603
Trigger-day E2, mean (SD), pg/ml	2451.88 (2197.88)	2769.22 (2413.64)	0.131	0.063	2700.69 (2396.70)	2642.27 (2278.14)	-0.024	0.777
Trigger-day P, mean (SD), ng/ml	1.07 (0.63)	1.12 (0.69)	0.064	0.364	1.10 (0.62)	1.12 (0.71)	0.031	0.712
Number of follicles, mean (SD)	6.89 (4.39)	7.45 (4.71)	0.119	0.098	7.43 (4.51)	7.33 (4.72)	-0.021	0.812
Number of mature follicles, mean (SD)	6.54 (4.29)	6.96 (4.47)	0.093	0.205	6.88 (4.42)	6.88 (4.53)	0.000	1.000
**Type of infertility** (n),%				0.842				0.655
Primary	291 (60.4)	168 (59.4)	-0.021		148 (57.4)	154 (59.7)	0.047	
Secondary	191 (39.6)	115 (40.6)	0.021		110 (42.6)	104 (40.3)	-0.047	
**Infertility diagnoses**								
Tubal factor (n),%				0.557				0.790
N0	286 (59.3)	161 (56.9)	-0.049		149 (57.8)	145 (56.2)	-0.031	
Yes	196 (40.7)	122 (43.1)	0.049		109 (42.2)	113 (43.8)	0.031	
Ovulation disorders (n),%				0.772				0.660
N0	398 (82.6)	230 (81.3)	-0.033		204 (79.1)	209 (81.0)	0.050	
Yes	84 (17.4)	53 (18.7)	0.033		54 (20.9)	49 (19.0)	-0.050	
Endometriosis (n),%				0.103				1.000
N0	475 (98.5)	273 (96.5)	-0.113		253 (98.1)	254 (98.4)	0.021	
Yes	7 (1.5)	10 (3.5)	0.113		5 (1.9)	4 (1.6)	-0.021	
POI (n),%				0.017				1.000
N0	391 (81.1)	249 (88.0)	0.211		224 (86.8)	225 (87.2)	0.012	
Yes	91 (18.9)	34 (12.0)	-0.211		34 (13.2)	33 (12.8)	-0.012	
Uterine factor (n),%				0.234				1.000
N0	473 (98.1)	273 (96.5)	-0.090		251 (97.3)	252 (97.7)	0.021	
Yes	9 (1.9)	10 (3.5)	0.090		7 (2.7)	6 (2.3)	-0.021	
Male factor (n),%				0.038				1.000
N0	376 (78.0)	201 (71.0)	-0.154		187 (72.5)	188 (72.9)	0.009	
Yes	106 (22.0)	82 (29.0)	0.154		71 (27.5)	70 (27.1)	-0.009	
Other causes (n),%				0.424				0.775
N0	422 (87.6)	254 (89.8)	0.073		232 (89.9)	229 (88.8)	-0.038	
Yes	60 (12.4)	29 (10.2)	-0.073		26 (10.1)	29 (11.2)	0.038	
Unexplained causes (n),%				0.096				0.515
N0	433 (89.8)	265 (93.6)	0.156		235 (91.1)	240 (93.0)	0.079	
Yes	49 (10.2)	18 (6.4)	-0.156		23 (8.9)	18 (7.0)	-0.079	
**Ovarian stimulation protocols (n),%**								
A(n),%				1.000				1.000
N0	481 (99.8)	283 (100.0)	0.057		258 (100.0)	258 (100.0)	0.000	
Yes	1 (0.2)	0 (0.0)	-0.057		0 (0.0)	0 (0.0)	0.000	
B(n),%				1.000				0.866
N0	446 (92.5)	262 (92.6)	0.002		238 (92.2)	240 (93.0)	0.030	
Yes	36 (7.5)	21 (7.4)	-0.002		20 (7.8)	18 (7.0)	-0.030	
C(n),%				0.146				1.000
N0	481 (99.8)	280 (98.9)	-0.083		257 (99.6)	258 (100.0)	0.038	
Yes	1 (0.2)	3 (1.1)	0.083		1 (0.4)	0 (0.0)	-0.038	
D(n),%				0.070				1.000
N0	415 (86.1)	257 (90.8)	0.163		234 (90.7)	233 (90.3)	-0.013	
Yes	67 (13.9)	26 (9.2)	-0.163		24 (9.3)	25 (9.7)	0.013	
E(n),%				0.052				0.857
N0	220 (45.6)	108 (38.2)	-0.154		102 (39.5)	99 (38.4)	-0.024	
Yes	262 (54.4)	175 (61.8)	0.154		156 (60.5)	159 (61.6)	0.024	
F(n),%				0.923				0.886
N0	435 (90.2)	254 (89.8)	-0.016		230 (89.1)	232 (89.9)	0.026	
Yes	47 (9.8)	29 (10.2)	0.016		28 (10.9)	26 (10.1)	-0.026	
G(n),%				0.657				1.000
N0	478 (99.2)	282 (99.6)	0.080		257 (99.6)	257 (99.6)	0.000	
Yes	4 (0.8)	1 (0.4)	-0.080		1 (0.4)	1 (0.4)	0.000	
H (n),%				0.217				1.000
N0	430 (89.2)	261 (92.2)	0.113		237 (91.9)	236 (91.5)	-0.014	
Yes	52 (10.8)	22 (7.8)	-0.113		21 (8.1)	22 (8.5)	0.014	
I(n),%				1.000				1.000
N0	470 (97.5)	276 (97.5)	0.001		251 (97.3)	251 (97.3)	0.000	
Yes	12 (2.5)	7 (2.5)	-0.001		7 (2.7)	7 (2.7)	0.000	
**Fertilization method(n),%**				0.505				0.795
IVF	331 (68.7)	199 (70.3)	0.036		178 (69.0)	182 (70.5)	0.034	
HALF-ICSI	42 (8.7)	18 (6.4)	-0.096		15 (5.8)	17 (6.6)	0.032	
ICSI	109 (22.6)	66 (23.3)	0.017		65 (25.2)	59 (22.9)	-0.055	
Fertilization rate	0.77 (0.27)	0.76 (0.26)	-0.057	0.443	0.77 (0.27)	0.76 (0.26)	-0.034	0.685

SD: standard deviation. SMD: standard mean difference. *P*: *p*-value

Before matching, Poisson regression analysis showed no effect of intravenous anesthetic drugs on fertilization rate (RR = 0.859, 95%CI:0.59 to 1.25, *P* = 0.422) (Table 2). After matching, the effect of intravenous anesthetic drugs remained unobvious (RR = 0.935, 95%CI:0.67 to 1.29, *P* = 0.681) (Table 2).

According to the results of univariate analyses presented in Table 3, variables including POI, ovarian stimulation protocols (C, E, and G), trigger-day E2, trigger-day P, number of mature follicles, fertilization method, and uterine factor were further examined in multivariable analyses, as shown in Table 4. Statistically significant differences were observed in the impact of ovarian stimulation protocols (C) (b = 0.323, t = 2.421, *p* = 0.016) and ovarian stimulation protocols (G) (b = 0.073, t = 2.028, *p* = 0.043) on the fertilization rate, as well as in the impact of the number of mature follicles (b = 0.008, t = 2.380, *p* = 0.018) and fertilization method (b = 0.063, t = 5.523, *p* = 0.000) on the fertilization rate (Table 4). The administration of intravenous anesthesia drugs did not demonstrate a significant impact on the rate of fertilized eggs, as indicated by the statistical analysis (b = 0.017, t = 0.813, *p* = 0.417) presented in Table 3.

**Table 2:** Results of the Poisson regression analysis

Poisson regression analysis	Before matching		After matching	
	RR (95%CI)	P	RR (95%CI)	P
Fertilization rate	0.859 (0.59 to 1.25)	0.422	0.935 (0.67 to 1.29)	0.681

**Table 3:** Univariate analyses

Variables	b	SE(b)	t	*p*
Number of IVF cycles	− 0.017	0.014	-1.164	0.245
Female age, mean	− 0.002	0.002	-1.152	0.250
Female BMI	− 0.004	0.003	-1.412	0.158
Male age	0.000	0.002	0.193	0.847
Duration of infertility	− 0.005	0.005	-1.067	0.286
Basal FSH	0.003	0.004	0.730	0.466
Basal LH	0.002	0.004	0.562	0.574
Basal E2	0.000	0.000	− 0.433	0.665
Basal AFC	0.001	0.001	0.654	0.513
Basal CA125	− 0.003	0.001	-1.817	0.070
Launch-day FSH	− 0.002	0.004	− 0.624	0.533
Launch-day LH	1.122E-5	0.004	0.003	0.997
Launch-day E2	9.928E-5	0.000	0.475	0.635
Launch-day AFC	-5.738E-5	0.001	− 0.050	0.960
Trigger-day LH	− 0.004	0.003	-1.306	0.192
Trigger-day E2	8.834E-6	0.000	2.055	0.040
Trigger-day P	0.036	0.015	2.430	0.015
Number of follicles	0.003	0.002	1.316	0.189
Number of mature follicles	0.008	0.002	3.470	0.001
Type of infertility	− 0.004	0.020	− 0.198	0.843
Tubal factor	0.016	0.020	0.799	0.424
Ovulation disorders	− 0.010	0.026	− 0.389	0.698
Endometriosis	− 0.080	0.067	-1.198	0.231
POI	− 0.054	0.026	-2.049	0.041
Uterine factor	− 0.054	0.026	-2.049	0.041
Male factor	0.016	0.023	0.717	0.473
Other causes	− 0.051	0.035	-1.479	0.140
Unexplained causes	0.010	0.031	0.318	0.751
Ovarian stimulation protocols (A)	0.235	0.272	0.861	0.389
Ovarian stimulation protocols (B)	0.011	0.037	0.289	0.773
Ovarian stimulation protocols (C)	− 0.320	0.136	-2.356	0.019
Ovarian stimulation protocols (D)	− 0.023	0.030	− 0.776	0.438
Ovarian stimulation protocols (E)	0.041	0.020	2.070	0.039
Ovarian stimulation protocols (F)	0.021	0.033	0.625	0.532
Ovarian stimulation protocols (G)	− 0.102	0.033	-3.114	0.002
Ovarian stimulation protocols (H)	0.135	0.122	1.107	0.269
Ovarian stimulation protocols (I)	− 0.052	0.063	− 0.821	0.412
Fertilization method	0.059	0.012	5.111	0.000
anesthesia	− 0.017	0.020	− 0.813	0.417

**Table 4:** Multivariable analyses

Variables	b	SE(b)	t	*p*
POI	0.004	0.030	0.118	0.906
Ovarian stimulation protocols (C)	− 0.323	0.133	-2.421	0.016
Ovarian stimulation protocols (E)	− 0.003	0.023	− 0.122	0.903
Ovarian stimulation protocols (G)	− 0.073	0.036	-2.028	0.043
Trigger-day E2	-4.436E-6	0.000	− 0.754	0.451
Trigger-day P	0.022	0.017	1.309	0.191
Number of mature follicles	0.008	0.003	2.380	0.018
Fertilization method	0.063	0.011	5.523	0.000
Uterine factor	− 0.032	0.024	-1.346	0.179

## Discussion

In clinical practice, an anesthetic modality should be set in subjects according to their willingness, pain tolerance, location of ovary and the number of oocytes. The current study showed that intravenous anesthetic drugs had no impact on the fertilization rate. Additionally considering that intravenous anesthesia could eliminate subjects’ pain and anxiety, related drugs might be recommended to females receiving oocyte retrieval in IVF.

Previous studies have found that the fertilization rate is significantly associated with the pregnancy outcome [6, 7]. The fertilization rate is a reliable biomarker of oocyte quality. There is also a strong relationship between the fertilization rate and the cumulative live birth rate (CLBR). Rehman et al.[8] have reported that subjects who have a lower fertilization rate achieve poorer pregnancy outcomes. Therefore, fertilization rate is used as a key laboratory indicator for the success or failure of IVF [9].

In this study, the intravenous propofol was used in the anesthesia group. As a popular intravenous drug, propofol functions fast, induces a smooth anesthesia, enables a rapid recovery, and minimizes postoperative events, such as nausea and vomiting. Propofol is also a lipid-soluble substance capable of entering the placenta. Anesthetic neurotoxicity in neonates and young children is a pressing concern [10] A large-scale retrospective study [11] in 2009 has found that children undergoing multiple exposures to anesthesia face an increased risk of neurocognitive defects. It reports that a single exposure to anesthesia before age 4 years is not associated with an increased risk of learning disability (LD), which may be observed in those with more exposures. Some studies [12, 13] have reported that chronic and repeated exposure of sedation medication, including benzodiazepines, opioids, propofol, and ketamine, causes neurodegeneration, suggesting that exposures and outcomes may have a dose-response and temporal association. So, repeated and prolonged anesthetic exposure should be avoided in neonates and young children. The present study, for the first time, revealed that after the brief exposure to propofol at the oocyte stage, propofol did not affect the quality of embryos and the IVF pregnancy outcomes.

Previous studies have investigated the effects of intravenous anaesthetics on pregnancy. In the studies by Ngamprasertwong et al.[14], an animal model of propofol-induced maternal fetal PK was successfully developed in pregnant sheep for the first time. The concentration of propofol in the fetus was much lower than that in ewes at mid-gestation. A study in the Europe has been conducted to investigate the anesthetics on learning at school age, finding that a brief duration of exposure is not associated with neurodevelopmental disabilities [15, 16]. Another study has verified that the safety of intravenous anesthetic drugs in cesarean section, suggesting that propofol has no effect on fetal growth and development [17]. Two meta-analyses [18, 19] have reported intravenous anesthetics, including propofol, fentanyl, and lidocaine, do not affect reproductive outcomes. Indirectly, these observations corroborate the conclusion of the present study.

Notably, oocyte retrieval is anxiety-provoking in the IVF treatment [20]. It may take multiple attempts to obtain a pregnancy. Severe pain may cause problems, such as prolonged operation, premature termination, and side effects during surgical procedures. These unpleasant experiences could results in excessive worry about IVF treatment [21] A research of Yoon Frederiksenet al. [22] has shown that about 7% of women feel distressed during oocyte retrieval. Combined with the findings in the present study, anesthetic measures should and could be taken to relieve the pain in women receiving oocyte retrieval.

The innovation of this study is that it adopts PSM to explore the effect of intravenous anesthetic drugs on fertilization rate for the first time. PSM can reduce inter-group differences and balance inter-group confounders. Meanwhile, there are some limitations to the study. First, this study is a single-center retrospective study with a small sample size, which may result in some deviations in the results. Even though PSM was used, unknown residual confounders could not be completely excluded.

## Conclusions

Intravenous anesthetic drugs (propofol) might exert no obvious impact on the fertilization rate and pregnancy outcomes in subjects receiving IVF. This finding is worthy of large-size and multi-center studies in the future.

### Electronic supplementary material

Below is the link to the electronic supplementary material.

Supplementary Material 1

## Data Availability

All data generated or analysed during this study are included in its supplementary information files. Please see the attachment.(data.xlsx).
